# Mutant NADH dehydrogenase subunit 4 gene delivery to mitochondria by targeting sequence-modified adeno-associated virus induces visual loss and optic atrophy in mice

**Published:** 2012-06-20

**Authors:** Hong Yu, Sacide S. Ozdemir, Rajeshwari D. Koilkonda, Tsung-Han Chou, Vittorio Porciatti, Vince Chiodo, Sanford L. Boye, William W. Hauswirth, Alfred S. Lewin, John Guy

**Affiliations:** 1 Bascom Palmer Eye Institute, University of Miami, Miller School of Medicine, Miami, FL; 2 Department of Ophthalmology, University of Florida, College of Medicine, Gainesville, FL; 3 Department of Molecular Genetics and Microbiology, University of Florida, College of Medicine, Gainesville, FL

## Abstract

**Purpose:**

Although mutated G11778A NADH ubiquinone oxidoreductase subunit 4 (*ND4*) mitochondrial DNA (mtDNA) is firmly linked to the blindness of Leber hereditary optic neuropathy (LHON), a bona fide animal model system with mutated mtDNA complex I subunits that would enable probing the pathogenesis of optic neuropathy and testing potential avenues for therapy has yet to be developed.

**Methods:**

The mutant human *ND4* gene with a guanine to adenine transition at position 11778 with an attached FLAG epitope under control of the mitochondrial heavy strand promoter (HSP) was inserted into a modified self-complementary (sc) adeno-associated virus (AAV) backbone. The HSP-*ND4FLAG *was directed toward the mitochondria by adding the 23 amino acid cytochrome oxidase subunit 8 (COX8) presequence fused in frame to the N-terminus of green fluorescent protein (GFP) into the AAV2 capsid open reading frame. The packaged scAAV-HSP mutant *ND4* was injected into the vitreous cavity of normal mice (OD). Contralateral eyes received scAAV-*GFP* (OS). Translocation and integration of mutant human *ND4* in mouse mitochondria were assessed with PCR, reverse transcription–polymerase chain reaction (RT–PCR), sequencing, immunoblotting, and immunohistochemistry. Visual function was monitored with serial pattern electroretinography (PERG) and in vivo structure with spectral domain optical coherence tomography (OCT). Animals were euthanized at 1 year and processed for light and transmission electron microscopy.

**Results:**

The PCR products of the mitochondrial and nuclear DNA extracted from infected retinas and optic nerves gave the expected 500 base pair bands. RT–PCR confirmed transcription of the mutant human *ND4* DNA in mice. DNA sequencing confirmed that the PCR and RT–PCR products were mutant human *ND4* (OD only). Immunoblotting revealed the expression of mutant ND4FLAG (OD only). Pattern electroretinograms showed a significant decrement in retinal ganglion cell function OD relative to OS at 1 month and 6 months after AAV injections. Spectral domain optical coherence tomography showed optic disc edema starting at 1 month post injection followed by optic nerve head atrophy with marked thinning of the inner retina at 1 year. Histopathology of optic nerve cross sections revealed reductions in the optic nerve diameters of OD versus OS where transmission electron microscopy revealed significant loss of optic nerve axons in mutant *ND4* injected eyes where some remaining axons were still in various stages of irreversible degeneration with electron dense aggregation. Electron lucent mitochondria accumulated in swollen axons where fusion of mitochondria was also evident.

**Conclusions:**

Due to the UGA codon at amino acid 16, mutant G11778A *ND4* was translated only in the mitochondria where its expression led to significant loss of visual function, loss of retinal ganglion cells, and optic nerve degeneration recapitulating the hallmarks of human LHON.

## Introduction

Leber hereditary optic neuropathy (LHON) is a maternally inherited disorder that usually results in loss of vision during the second and third decades of life. The guanine to adenine transition at nucleotide 11778 in mitochondrial DNA (mtDNA) in the gene specifying the nicotinamide-adenine dinucleotide (NADH) dehydrogenase subunit 4 (*ND4*) of complex I causes half the cases [1]. This mutation results in an arginine to histidine substitution at amino acid 340 [2]. Of all diseases caused by mutated mitochondria, LHON is the most common [3]. Complex I contains seven subunits encoded by mtDNA that are synthesized within the organelle and 35 subunits encoded by nuclear DNA that are imported from the cytoplasm [4]. Mutations in two additional mtDNA encoded complex I subunits, *ND1* and *ND6*, have also been associated with LHON. Together with *ND4*, they are responsible for most of the cases of LHON [5,6]. The *ND4* mutation is the most severe, showing little propensity for spontaneous recovery of vision, thus making this mutation an attractive target for intervention.

Common to most mitochondrial encoded proteins, mitochondrial *ND4* can be translated only in the mitochondria and not on cytoplasmic ribosomes, because the TGA codon at positions 16 and 24 that code for tryptophan in the mitochondria are stop codons in the nuclear genetic code. Since there was no way to introduce DNA directly into the mitochondria of a live animal, we had previously knocked down expression of a critical nuclear-encoded complex I subunit, NADH ubiquinone oxidoreductase subunit NDUFA1 (NDUFA1), to induce optic neuropathy in the mouse [7]. Several years later, we added a mitochondrial targeting sequence (MTS) to the N-terminus of a nuclear-encoded version of the mutant human *ND4* subunit gene that induced a phenotype in the mouse with remarkable similarity to the human LHON disorder [8].

In this report, we describe redirection of the adeno-associated virus (AAV) virion to mitochondria by adding an MTS to the viral outer capsid to deliver the mutant G11778A human *ND4* gene in the mitochondrial genetic code to the visual system of mice where the mutant *ND4* allele induced retinal ganglion cell (RGC) dysfunction and optic atrophy. AAV is a single-stranded DNA parvovirus with a 4.7 kb genome and a particle diameter of 21 nm. The AAV genome consists of two genes, replicase (*REP*) and capsid (*CAP*), which encode the non-structural REP proteins (REP78, REP68, REP52, and REP40) and the capsid proteins (VP1, VP2, and VP3), respectively. Flanking these two genes are the inverted terminal repeat (ITR) sequences that provide all the cis-acting sequences required for replication, packaging, and integration [9]. The VP2 capsid protein of AAV serotype 2 can tolerate large peptide insertions at the N-terminus without loss of infectivity [10]. However, VP2 is not essential for the infectivity of AAV, and in the AAV capsid only three of 60 subunits are VP2.

## Methods

### Plasmids

To construct the fusion gene containing the mitochondrial promoter, human wild-type *ND4* and epitope tag, mitochondrial DNA was extracted from human cells. Using the high fidelity of *pfu* Turbo DNA polymerase (Stratagene, Santa Clara, CA), the 1.4 kb mitochondrial encoded *ND4* gene was cloned into the Topo TA cloning vector using a kit according to the manufacturer's directions (Invitrogen, Carlsbad, CA). A final extension step using Taq DNA polymerase was also performed. The QuikChange in vitro mutagenesis kit (Stratagene) was used to add the FLAG epitope tag with appended AGA termination codon to the 3′ end of the open reading frame of the *ND4* gene to obtain *ND4FLAG*. Base deletions and substitutions in the reading frame *ND4FLAG* were corrected using the QuikChange in vitro mutagenesis kit (Stratagene). Human wild-type *ND4* fused in frame with FLAG was cloned into pCDNA3 and used as a template to generate mutant human *ND4* with a substitution of A for G at nucleotide 1019 with site-directed mutagenesis (Quikchange II XL site-directed mutagenesis kit; Stratagene). After confirmation with sequencing, the generated mutant *ND4G1019AFLAG* was then cloned into self-complementary AAV backbones under the control of the mitochondrial heavy strand promoter (HSP). The parent pTR-UF plasmid was digested with Xba I and BamHI to remove the green fluorescent protein (*GFP*) and neomycin resistance (*neoR*) genes. The Topo plasmid containing the mitochondrial *ND4FLAG* was also digested with Xba I and BamHI for directional cloning into the similarly digested recipient pTR-UF11 plasmid. The *ND4FLAG* insert was then ligated into the recipient pTR-UF11 plasmid. This gave the desired plasmid designated as pTR-UF11-m*ND4FLAG*. Next, the hybrid CMV enhancer and chicken β-actin promoter elements (used to drive gene expression in the nucleus) was removed from pTR-UF11-*ND4FLAG* using KpnI and Xba I digests. We purchased complementary oligonucleotides (Invitrogen) containing the mitochondrial HSP, sequence 5′-TAA CCC CAT ACC CCG AAC CAA CCA AAC CCC AAA GAC AC-3′ with added linkers containing the KpnI restriction site at the 5′ end and Xba I at the 3′ end of HSP. The annealed double-stranded HSP oligonucleotide was directionally ligated into the pTR-UF11-*ND4FLAG *to give plasmid pTR-UF11-HSP-*ND4FLAG*. We then inserted *HSP-ND4FLAG* or HSP mutant*ND4FLAG* into a self-complementary (sc) AAV backbone. The parent SC-trs-SB-smCBA-*hGFP* plasmid was digested with KpnI and BamHI to remove the hybrid CMV enhancer and small chicken β-actin promoter elements as well as GFP. The insert containing HSP-*ND4FLAG* was digested out of pTR-UF11-*HSP-ND4FLAG* using restriction enzymes KpnI and Xba I, and was then ligated into the recipient scAAV backbone generating the construct sc-HSP-*ND4FLAG* containing flanking ITRs that provide all the* cis*-acting sequence required for replicating, packaging, and integrating the AAV vector.

To generate an AAV directed toward the mitochondria, we added a mitochondrial targeting sequence (MTS) to the open reading frame of VP2, one of the three capsid proteins that constitute an AAV virion. This MTS-targeted VP2 was generated by linking the 23 amino acid MTS presequence of COX8, 5′ATG TCC GTC CTG ACG CCG CTG CTG CTG CGG GGC TTG ACA GGC TCG GCC CGG CGG CTC CCA GTG CCG CGC GCC AA- 3′ in frame to *GFP*. Eag I linkers were added to the COX8GFP insert. The parent VP2 plasmid with an Eag I site inserted at residue 138 within the VP1-VP2 overlap region was digested with Eag I. The *COX8GFP* was ligated into the mutant VP2 parent plasmid. This created the plasmid designated *COX8GFP* VP2. Construction of the mutant VP2 parent plasmid and the GFP-containing VP2 mutant plasmid without the MTS has previously been described [10].

### Adeno-associated viruses

Self-complementary AAV2/sc-HSP-*ND4FLAG*, *HSP* mutant*ND4FLAG* or a control scAAV2-*GFP* was produced with the plasmid cotransfection method [11]. The plasmids were packaged with the *COX8GFP* VP2 (mutant or wild-type human *ND4*) using the PXX6 helper plasmid (threefold excess) into the AAV2 recombinant virus by transfection into human 293 cells with standard procedures. Briefly, the crude iodixanol fractions were purified using the Pharmacia AKTA FPLC system, the virus was then eluted from the column with 215 mM NaCl, pH 8.0, and the rAAV peak was collected. rAAV-containing fractions were then concentrated and buffer exchanged in Alcon BSS with 0.014% Tween-20, with a Biomax 100 K concentrator (Millipore, Billerica, MA). The virus titer was then measured for DNase-resistant viral genomes with real-time PCR relative to a standard. Finally, the purity of the virus was validated with silver-stained sodium dodecyl sulfate–PAGE, assayed for sterility and lack of endotoxin, and then aliquoted and stored at −80 °C.

### Cell culture and infection

Human embryonal kidney cells (293T) or mouse embryonal fibroblasts infected with AAV vectors were cultured in complete high-glucose medium (DMEM). The endogenous fluorescence of *COX8GFP* VP2 scAAV-infected cells was observed in live cells. The cells cultured on plastic chamber slides were reacted with MitoTracker Green according to the manufacturer's specifications (Invitrogen) and then fixed in 4% paraformaldehyde for examination with a confocal microscope.

### Animals

All animal procedures were performed in accordance with the National Institutes of Health Guide for Care and Use of Laboratory Animals and the ARVO Statement for the Use of Animals in Ophthalmic and Vision Research and approved by the UM IACUC. For the intraocular injection of recombinant AAV, DBA/1J mice were sedated by inhalation with 1.5% to 2% isoflurane. A local anesthetic (proparacaine HCl) was applied topically to the cornea, and then a 32-gauge needle attached to a Hamilton syringe was inserted through the pars plana. One microliter of MTS-targeted scAAV2 containing mutant or wild-type human *ND4* DNA (1.01×10^11^ VG/ml), 2.19×10^12^ VG/ml) was injected in the right eye and scAAV-*GFP* (1.03×10^12^ VG/ml) injected into the left eye. The animals were euthanized at certain time points post injection.

### Electrophysiology

In brief, mice were weighed and anesthetized with intraperitoneal injections of a mixture of ketamine (80 mg/kg bodyweight) and xylazine (10 mg/kg bodyweight) and were restrained with a bite bar and a nose holder that allowed unobstructed vision. The animals were kept at a constant body temperature of 37.6 °C with a feedback-controlled heating pad. In these conditions, the eyes were wide open and in a stable position with undilated pupils pointing laterally and upward. The electroretinogram electrode had a diameter of 0.25 mm, and was made of silver wire configured to a semicircular loop with a 2-mm radius, which was placed on the corneal surface with a micromanipulator and positioned to encircle the pupil without limiting the field of view. Reference and ground electrodes were stainless-steel needles inserted under the skin of the scalp and tail, respectively. After the mice were set on the stage and before responses were recorded, a small drop of balanced saline was topically applied on the cornea to prevent drying. A visual stimulus of contrast-reversing bars (field area, 50°×58°; mean luminance, 50 cd/m^2^; spatial frequency, 0.05 cycles/deg; contrast, 100%; and temporal frequency, 1 Hz) was aligned with the projection of the pupil at a distance of 20 cm. The eyes were not refracted for the viewing distance, because the mouse eye has a large depth of focus due to the pinhole pupil. Retinal signals were amplified (10,000 fold) and band-pass filtered (1–30 Hz). Three consecutive responses to 600 contrast reversals each were recorded. The responses were superimposed to check for consistency and then averaged. Pattern electroretinography (PERG) is a light-adapted response. To have a corresponding index of outer retinal function, light-adapted flash electroretinography was also recorded with undilated pupils in response to strobe flashes of 20 cd/m^2^/s superimposed on a steady background light of 12 cd/m^2^ and presented within a Ganzfeld bowl. Under these conditions, rod activity is largely suppressed while cone activity is minimally suppressed. To prevent bias in evaluating the peaks and troughs of PERG waveforms with reduced amplitude, the root-mean-square (RMS) voltage of the PERG signal was calculated. Response amplitude was defined as the RMS voltage over a time epoch of 0 to 350 ms after each contrast reversal according to the equation [VRMS=√ ((Σ v_i_^2^)/n], where VRMS is the amplitude, v_i_^2^ is the voltage at each sampled time point, and n is the number of sampled points. RMS evaluation was automatically performed using a macro written in Sigma Plot language (Sigmaplot 11.2, Systat Software Inc., San Jose, CA).

### In vivo imaging

In vivo high-resolution imaging of the living mouse retina was performed with spectral domain optical coherence tomography (OCT), as previously described [12]. Briefly, ketamine/xylazine anesthetized mice were restrained in a mounting tube that was fixed on a platform. Rasterscans—typically measuring 512×128 (horizontal x vertical) and 1024×64 depth scan patterns, with the fast scan in the horizontal direction—were performed for each eye. The scan length was approximately 32° for imaging mouse retinas. Acquiring high-quality OCT images took approximately 5 min for each mouse.

### Polymerase chain reaction and reverse transcriptase–polymerase chain reaction

Mitochondrial and nuclear fractions were isolated from pooled ocular tissues (n=6 mice). DNA and RNA were extracted respectively from each fraction with the DNeasy Blood and Tissue Kit (Qiagen) and the RNeasy Protect Mini Kit (Qiagen). Reverse transcription was performed with the IScript One-Step RT–PCR kit (Bio-Rad). PCR was performed using primers hND4F1: 5′-ATG CTA AAA CTA ATC GTC C-3′ and hND4R1403: 5′-CTC TTG TCA TCG TCG TCC-3′ for full-length ND4FLAG of 1.4 kb; hND4F5415: 5′-TGC ATA CTC TTC AAT CAG CC-3′ and hND4R1403: 5′-CTC TTG TCA TCG TCG TCC-3′ for 500 bp ND4FLAG. The resultant PCR products were sequenced with primer hND4F5415: 5′-TGC ATA CTC TTC AAT CAG CC-3′ or hND4F1: 5′-ATG CTA AAA CTA ATC GTC C-3′.

### Histology and ultrastructure

At the appropriate time points, all mice inoculated with the AAV vectors were injected with Euthasol Solution. They were then perfused by cardiac puncture with phosphate buffered solution (PBS; Na_2_HPO_4 _10.9 g, NaH_2_PO_4_ 3.2 g, NaCl 90 g, H_2_O 1000 ml; pH to 7.4) and then with fixative consisting of 4% paraformaldehyde in 0.1 M PBS buffer (pH 7.4). Whole retinas or longitudinal retinal sections were used for immunofluorescence. The following antibodies were used: Cy3-conjugated FLAG (1:100; Sigma), porin (1:100; Abcam), Thy1.2 (1:1500; Sigma), GFP (1:100; Sigma), NADH dehydrogenase subunit 4 (1:480; Abcam), and secondary antibodies that included antirat 488, antimouse Cy5, antirabbit 647, antirabbit Cy3 (1:600; Invitrogen), or antimouse immunoglobulin conjugated to immunogold (Ted Pella, Redding, CA) that was enhanced with a kit according to the manufacturer’s specifications (Ted Pella). Images were visualized with a Leica TCS (Buffalo Grove, IL) SP5 confocal microscope.

For electron microscopy, infected cultured cells or the eyes and optic nerves of infected rodents that were dissected after euthanasia and perfusion with fixative (4% paraformaldehyde, 2.5% glutaraldehyde) were further processed by immersion fixation in 2.5% glutaraldehyde, postfixed in 1% osmium tetroxide, 0.1 M sodium cacodylate-HCl buffer (pH 7.4), and 7% sucrose in the cold, and then dehydrated through an ethanol series to propylene oxide, infiltrated, and embedded in epoxy resin that was polymerized at 60 °C overnight. For immunocytochemistry, tissue specimens were postfixed in 5.0% acrolein, 0.1 M sodium cacodylate-HCl buffer (pH 7.4), and 7% sucrose and then dehydrated through an ethanol series and embedded in LR White (Ted Pella) that was polymerized at 50 °C overnight. Semithin longitudinal sections (0.5 µm) of the optic nerve head and retrobulbar nerve were stained with toluidine blue for light microscopic examination. Ultrathin sections (90 nm) were placed on nickel grids for immunocytochemistry with anti-FLAG antibodies or GFP antibodies counterstained by secondary antibodies conjugated to 5-nm immunogold. The gold particles were then amplified with a silver enhancement kit (Ted Pella). Examinations were made with a Hitachi H-7600 (Tokyo, Japan) with a tilting stage for three-dimensional rendering of the collected images or an H-7650 transmission electron microscope, operating at 200 or 80 kV, respectively.

### Immunoblotting

For western blot analysis, sonicated proteins from total cellular lysates or mitochondrial pellets obtained from infected ocular tissues (n=10 mice) were electrophoresed through a 10% polyacrylamide gel and electrotransferred to a polyvinylidene fluoride membrane. The membrane was immunostained with antibodies against ND4 (1:200; Santa Cruz, Santa Cruz, CA), FLAG (1:2,000; Sigma), or NDUFS4 (1:1,000; Mitosciences, Eugene, OR) followed by either antimouse or antirabbit horseradish peroxidase (HRP)-conjugated antibodies. Immune complexes were detected using ECL reagents (HRP-conjugated antibodies) and a FUJIFilm Imaging system (Valhalla, NY).

### Optic nerve areas and axon counts

One year after the viral gene injections, the optic nerves were dissected from 1 mm behind the globe to the optic chiasm. After the specimens were prepared, toluidine blue stained images were collected using an Olympus IX50 microscope, equipped with an Olympus DP7e CCD camera (Olympus America, Melville, NY). The optic nerve cross sections were manually traced for area measurements by an observer masked to the treatment. For the axon counts, 15 transmission electron micrographs were photographed at low magnification (2,500×) for each mutant ND4 injected optic nerve specimen and five micrographs for controls injected with AAV-GFP. The number of axons was then manually counted by an observer masked to the treatment agent. Axons were identified by the internalized neurotubules and axolemma surrounded by the electron-dense myelin sheath. Axons counts were expressed per mm2.

### Statistical analysis

Values were expressed as means±standard deviation (SD). Data were analyzed via the Student *t*-test for paired data. p values of <0.05 were considered significant, and p<0.01 was highly significant.

## Results

### Mitochondrial targeting sequence modified AAV does not lose infectivity

**Figure 1:**
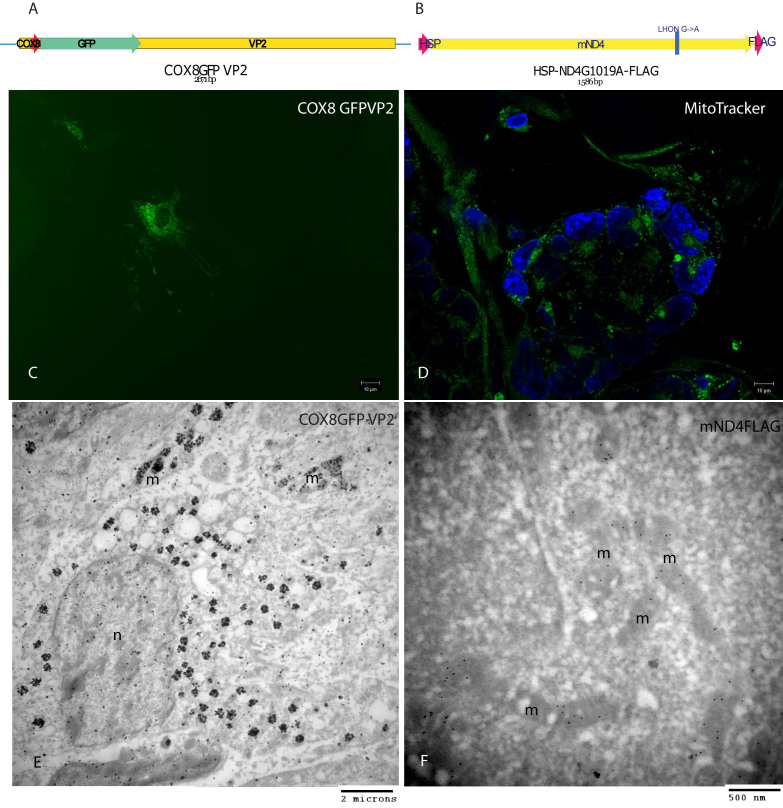
MTS AAV-capsid localizes within mitochondria where the delivered wild-type human *ND4* is translated in cultured cells. **A**: Illustration of the COX8 MTS fused in frame with *GFP* and inserted into the VP2 capsid of AAV. **B**: Illustration of the mitochondrial heavy strand promoter (HSP) driving expression of the mitochondrial mutant human ND4 subunit of complex I to which is appended a FLAG epitope. **C**: Fluorescence microscopy of live cultured cells infected with the *COX8GFP* VP2 MTS AAV revealed punctate and perinuclear expression of GFP suggestive of mitochondrial localization. **D**: MitoTracker Green staining of mitochondria surrounds nuclei labeled with DAPI. **E**: Transmission electron microscopy shows silver enhanced GFP immunogold inside mitochondria as well as within the nucleus. **F**: ND4FLAG immunogold is evident only within the mitochondria. Abbreviations m=mitochondria and n=nucleus.

We directed the AAV virion toward the mitochondria by adding the 23 amino acid cytochrome oxidase subunit 8 (COX8) presequence fused in frame to the N-terminus of GFP into the AAV2 capsid open reading frame (Figure 1A). To direct expression of exogenous wild-type human *ND4* in the organelle, we linked a mitochondrial promoter, the HSP, to the N-terminus of either wild-type human *ND4* or G11778A *ND4* cDNA (Figure 1B) to a scAAV backbone that contained AAV2 inverted terminal repeats (ITRs). For immunodetection, we linked a FLAG epitope to the C-terminus of G11778A *ND4*. The COX8 GFP coding sequence was ligated into the AAV capsid VP2 gene at a unique *EAGI* site at residue 138. This insertion did not substantially reduce infectivity of the recombinant AAV [13], as we achieved relatively high titers (2.2×10^12^ vg/ml) for this mitochondria-targeted AAV.

### Mitochondrial targeting sequence AAV-capsid localizes within mitochondria where the delivered human ND4 is translated in cultured cells

Fluorescence microscopy of live cultured cells infected with the *COX8GFP* VP2 MTS AAV revealed punctate and perinuclear expression of GFP (Figure 1C), suggestive of mitochondrial localization. Cells stained with a bona fide mitochondrial marker MitoTracker Green revealed a similar cellular distribution of mitochondria (Figure 1D). To determine if the MTS AAV virion entered the mitochondria, we used transmission electron microscopy where GFP labeled by silver enhanced immunogold decorated the interior of the organelle (Figure 1E). GFP immunogold was also seen within the nucleus where the AAV typically delivers its DNA. Control cells labeled only with the secondary antibody showed a background of approximately one to two gold particles per micrograph. In addition, wild-type ND4FLAG labeled by immunogold was seen only within the organelle (Figure 1F). Thus, it appears that, at least in cultured cells, a mitochondrial-encoded gene can be delivered to the interior of the organelle where the protein specified by the gene is translated.

### Mutant human G11778A ND4 transmission in mice

Next, we tested for MTS AAV DNA in the mouse visual system. Here the right eyes of the mice received injections into the vitreous body of sc-HSP-*ND4FLAG* or sc-HSP mutant*ND4FLAG* packaged by *COX8GFP* VP2 AAV. Injections of scAAV-*GFP* into the left eyes served as controls. To determine if the mitochondria-associated virus transferred mutant human *ND4FLAG* DNA, mitochondrial-enriched, cytoplasmic, and nuclear DNA fractions were extracted from the retinas and optic nerves of mice a week after intraocular viral injections. PCR for *ND4FLAG* gave the expected 500 kb band for the retinas and optic nerves of COX8 targeted G11778A *ND4FLAG*. The corresponding DNA sequences and alignment to wild-type human and mouse ND4 confirmed that the PCR products were indeed mutant human G11778A *ND4*, further supporting that exogenous *ND4* was imported into retinal and optic nerve mitochondria by a mitochondria-targeted AAV.

### Mutant human G11778A ND4 transcription in mice

**Figure 2:**
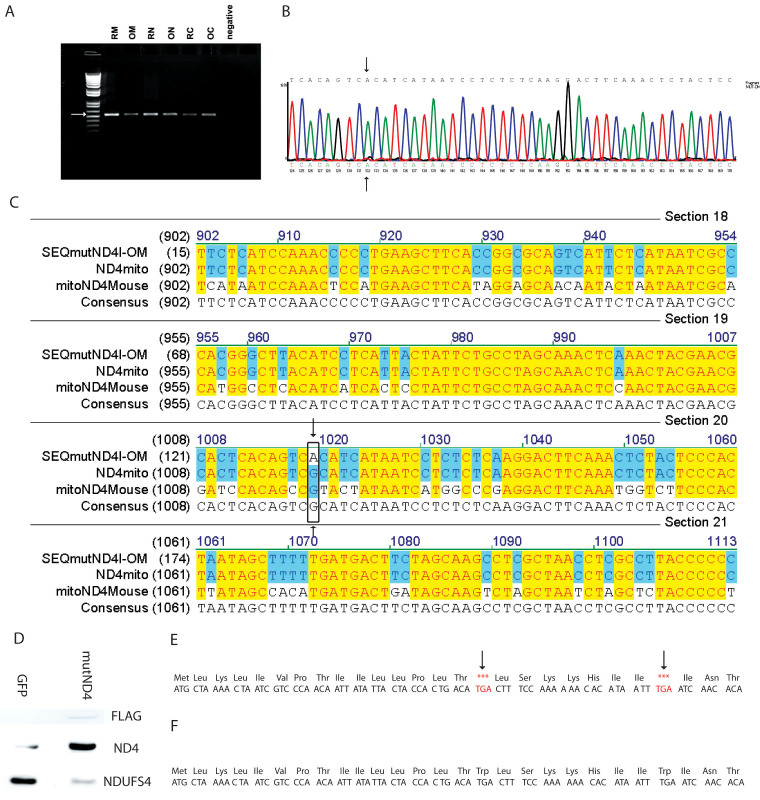
Mutant human G11778A *ND4* transmission in mice. **A**: RT–PCR of RNA extracted from retinal mitochondria (RM), optic nerve mitochondria (OM), retinal nuclear (RN), or optic nerve nuclear (ON) debris, retinal cytoplasm (RC), and optic nerve cytoplasmic (OC) fractions of experimental right eyes had the expected 500 bp band for *ND4FLAG *that was absent in RNA extracted from control left eyes infected with scAAV-*GFP*. **B**: A sequencing chromatograph shows the corresponding DNA sequence to be that of the mutant human *ND4* where the base adenine (A; arrows) has replaced guanine (G). **C**: One of the sequences, SEQmutND41-OM, is aligned to the wild-type human ND4 (ND4mito) showing this G to A transition (arrows). It also reveals the sequence of the mouse *ND4* (mitoND4mouse) confirmed that the PCR products were indeed mutant human G11778A *ND4*, further supporting that exogenous *ND4* was imported into retinal and optic nerve mitochondria by a mitochondria-targeted AAV where it was transcribed. **D**: Immunoblotting of isolated optic nerve and retinal mitochondria showed that the MTS-targeted AAV directed the synthesis of mutant human *ND4FLAG* in the experimental eyes, but the control eyes injected with GFP were negative for FLAG. ND4 was overexpressed in experimental eyes relative to the endogenous ND4 of GFP injected control eyes. Expression of NDUFS4, a nuclear encoded complex I subunit, is shown for housekeeping. **E**: Amino acid sequence of *ND4* with the start methionine (met) shows that the TGA codon is a termination sequence for protein synthesis in the cytoplasm, but specifies the amino acid tryptophan for synthesis within the mitochondrial ribosomes. **F**: Illustrating that full-length ND4 with 340 amino acids can be expressed only within mitochondria.

To determine if the mitochondria-associated virus transfer of mutant human *ND4FLAG* DNA to the organelle was transcribed, mitochondrial-enriched, cytoplasmic, and nuclear RNA fractions were extracted from the retinas and optic nerves of mice a week after intraocular viral injections. RT–PCR for *ND4FLAG *gave the expected 500 kb band for the retinas and optic nerves of MTS-targeted G11778A *ND4FLAG* (Figure 2A). DNase I–treated control samples gave no RT–PCR product. The corresponding DNA sequences (Figure 2B) and alignment to wild-type human and mouse *ND4 *confirmed that the RT–PCR products were indeed mutant human G11778A *ND4* (Figure 2C), supporting that exogenous *ND4* was translated in retinal and optic nerve mitochondria following gene delivery by a mitochondria-targeted AAV.

### Mutant human G11778A ND4 translation in mice

Immunoblotting of isolated optic nerve and retinal mitochondrial proteins showed that the MTS-targeted AAV directed the synthesis of mutant human *ND4FLAG* detected with an anti-FLAG antibody in experimental eyes but not in control eyes injected with scAAV-*GFP* (Figure 2D). In mutant *ND4* injected eyes, ND4 (52 kDa) was overexpressed relative to the control eyes injected with *GFP*. The housekeeping NDUFS4 (18 kDa) protein is shown for comparison. Although *ND4* cDNA and RNA were detected in the nucleus and cytoplasm, translation of *ND4* can occur only within the mitochondria. This is due to the partially different genetic codes, where the TGA codon is a termination sequence for protein synthesis in the nucleus and cytoplasm (Figure 2E), but specifies the amino acid tryptophan within the mitochondria (Figure 2F).

### Wild-type human ND4FLAG expression in retinal ganglion cells does not cause visual loss

**Figure 3:**
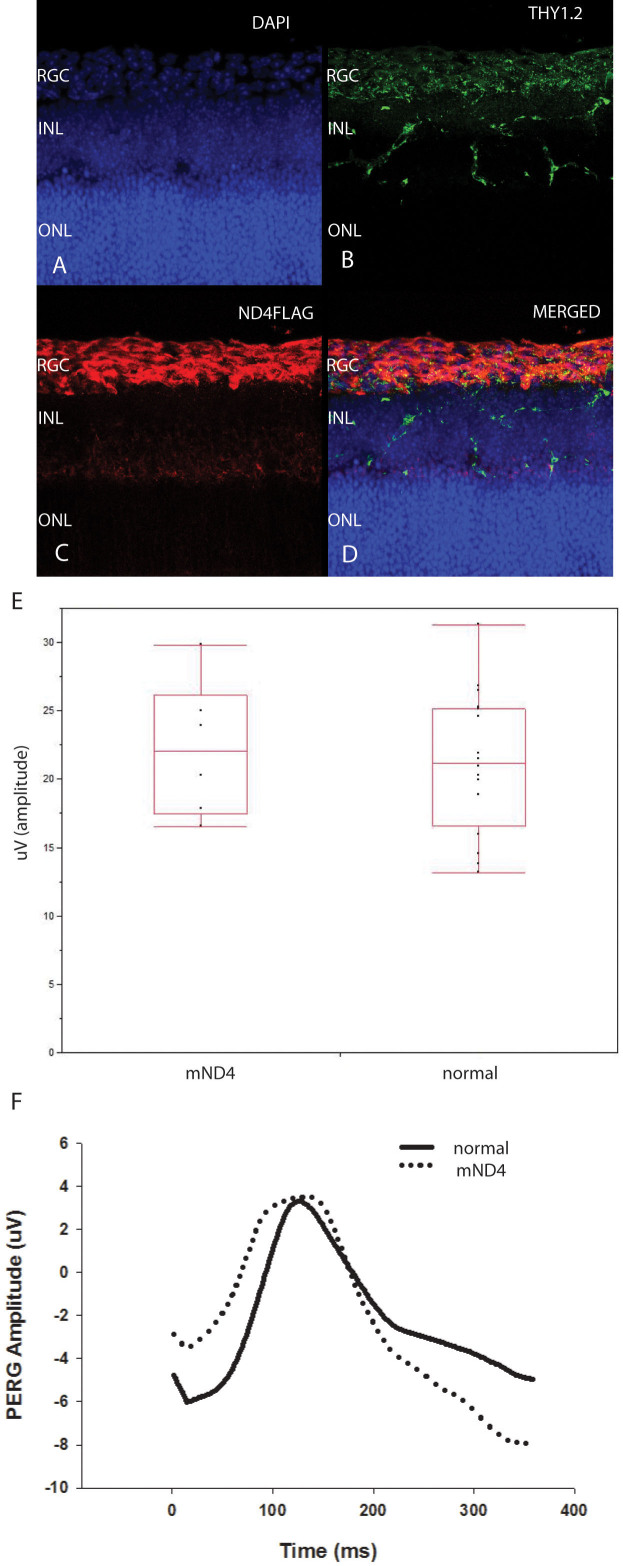
Wild-type human ND4 expression in RGCs does not cause visual loss in mice. Confocal immunofluorescence micrographs show DAPI stained nuclear layers of the murine retina infected 7 weeks earlier with MTS scAAV containing wild-type normal human *ND4FLAG* (**A**). The RGC layer and dendrites extending into the inner nuclear layer (INL) are labeled with an antibody against Thy1.2. The outer nuclear layer (ONL) is not stained by Thy1.2 (**B**). RGCs expressing human ND4FLAG are labeled by an anti-FLAG antibody (**C**) that colocalizes to Thy1.2 and has a perinuclear distribution characteristic of mitochondria surrounding the DAPI-stained RGC nuclei (**D**). A scatterplot (**E**) and a plot of representative PERG waveform (**F**) show that 7 weeks after AAV injections there were no differences in amplitude between the eyes injected with wild-type human mitochondrial (m) *ND4* relative to the eyes that received no ocular gene injections.

Immunohistochemistry of the retinas of eyes injected with the MTS AAV containing wild-type human G11778G *ND4* cDNA showed that in the RGC layer (Figure 3A) where RGCs were labeled by an antibody against Thy1.2 (Figure 3B), mutant or wild-type human ND4FLAG expression (Figure 3C) colocalized to RGCs (Figure 3D) whose axons comprised the optic nerve. Our next step was to determine that expression of wild-type human ND4 would not cause visual loss in mice. For this, we tested the visual function of eyes of animals (n=6) injected with wild-type *ND4* packaged with MTS-targeted AAV. Seven weeks after AAV injections, PERGs, a sensitive measure of RGC and visual function, showed no differences in amplitude between eyes injected with wild-type human mitochondrial (m) *ND4* (22.2±2.1 uV; mean±standard error) relative to uninjected eyes (21.2±1.3, n=16; Figure 3E-F).

### Mutant human G11778A ND4 causes visual loss in mice

**Figure 4:**
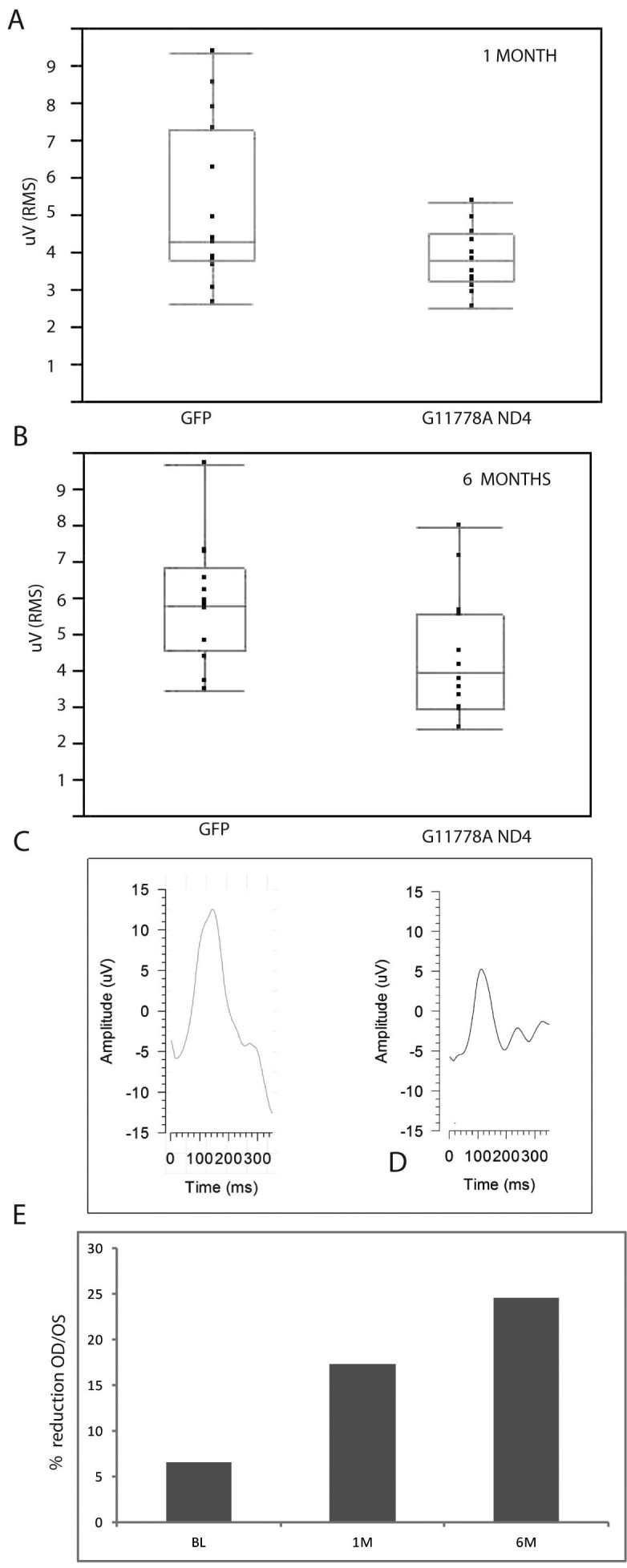
Mutant human ND4 expression induces visual loss in mice. **A**: At 1 month post injection, a scatterplot of a root-mean-square (RMS) PERG amplitude shows a significant loss of visual function of the right eyes injected with the mutant human *ND4* packaged with the MTS scAAV relative to the left eyes injected with *GFP* packaged with standard scAAV (p=0.0352). **B**: Six months post injection, this decrement between experimental and control eyes was even more significant (p=0.0182). **C**: Representative waveforms show normal amplitude 6 months after scAAV-*GFP* injection but loss (**D**) of PERG signal with MTS scAAV mutant *ND4* injection. E: A barplot shows an increasing reduction of the ratio of the right eye to left eye amplitudes with 1-month (1M) and 6-month (6M) intervals post injection relative to baseline values (BL) obtained before AAV injections.

In contrast, starting at 1 month post injection, PERGs of the right eyes injected with the mutant human *ND4* packaged with the MTS scAAV showed a significant decrement in RGC function (3.96±0.45 μV RMS; mean±standard error) relative to the left eyes injected with scAAV-*GFP* packaged with standard scAAV2 (5.16±0.45 μV RMS), p<0.04 (n=15 mice; Figure 4A). Six months post injection, this decrement between the experimental (4.43±0.45 μV RMS) and control eyes (5.85±0.46 μV RMS) was maintained, p<0.02 (Figure 4B-E). In a comparison of the 1-month PERGs and 6-month PERGs of the right eyes, we found no statistically significant difference (p=0.4). Similarly the left eyes injected with AAV-*GFP* had no statistically significant difference in the PERGs at 1 month relative to 6 months post injection (p=0.36). Thus, as also seen in patients with LHON, our mice with expression of G11778A mutant human *ND4* had an initial drop in visual function with little propensity for spontaneous recovery.

### In vivo imaging of early optic disc edema and later optic nerve head atrophy

**Figure 5:**
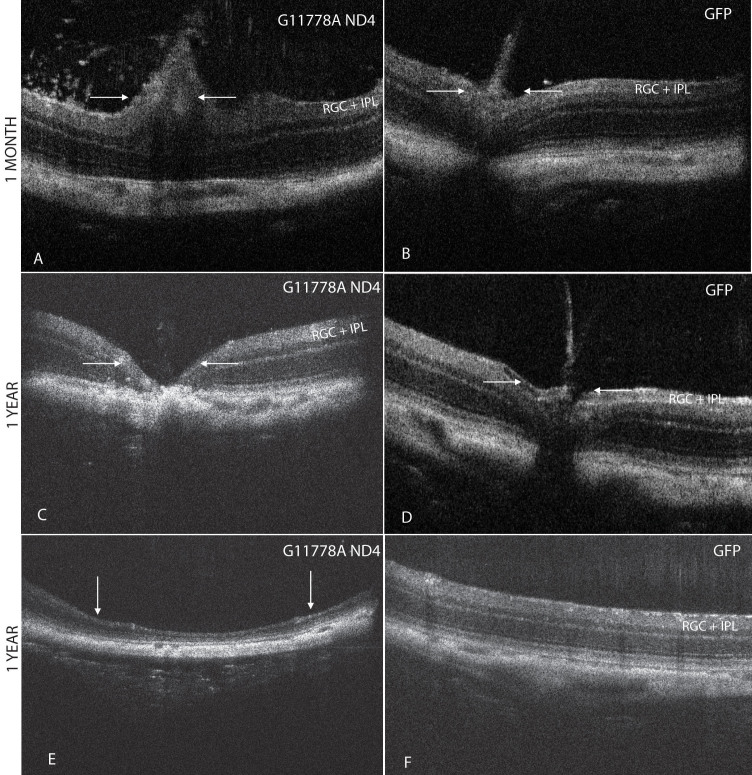
SD-OCT imaging of optic disc edema and optic atrophy. **A**: Spectral domain optical coherence tomography (SD-OCT) of right eyes injected with the mutant human *ND4* packaged with the MTS scAAV revealed swelling of the optic nerve head (arrows) commencing at 1 month post injection. A focal increase in the thickness of the RGC and the inner plexiform layer (IPL) is apparent just to the right of the swollen optic nerve head. **B**: The control eye injected with scAAV-GFP showed the normal anatomy of the mouse optic nerve head. **C**: One year post injection, optic nerve head atrophy was apparent in the mutant ND4 injected eyes. **D**: The contralateral GFP injected control eyes maintained normal optic nerve head anatomy. **E**: One year post injection, focal thinning with loss of the inner retina was also apparent in an experimental eye, but this finding was not seen in any of the control eyes (**F**). RGC=retinal ganglion cell layer; IPL=inner plexiform layer.

Spectral domain optical coherence tomography (SD-OCT) of right eyes injected with the mutant human *ND4* packaged with the MTS scAAV revealed swelling of the optic nerve head starting at 1 month post injection (Figure 5A) relative to the contralateral control eye injected with scAAV-*GFP* (Figure 5B). One year later, optic nerve head atrophy (Figure 5C) was apparent in the mutant *ND4* injected eyes but not in the *GFP* controls (Figure 5D). At this time point, marked focal thinning of the inner retina was also apparent (Figure 5E) in an experimental eye but not observed in control eyes (Figure 5F).

### Postmortem histopathology of retinal ganglion cell loss

**Figure 6:**
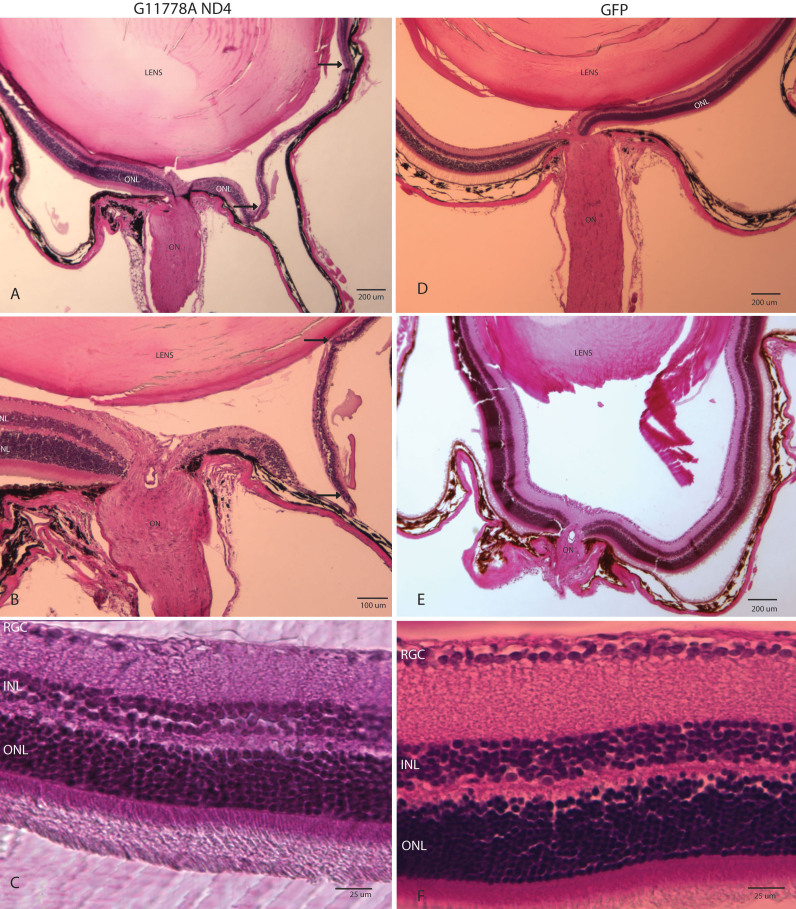
Histopathology of RGC loss. Light microscopy of the eyes of mice euthanized a year post AAV injections confirmed the OCT findings of inner retinal loss induced by injection of the mutant *ND4* MTS AAV showing marked focal thinning of the inner retina at low (**A**) and higher (**B**) magnifications. **C**: While experimental eyes of other animals did not exhibit such severe inner retinal atrophy, the more characteristic loss of cells in the ganglion cell layer was evident. Control eyes injected with scAAV-*GFP* showed no loss of the inner retina layers (**D** and** E**) or cells in the RGC layer (**F**). ON=optic nerve, RGC=retinal ganglion cell layer, INL=inner nuclear layer, and ONL=outer nuclear layer.

Light microscopy of the eyes of mice euthanized a year post AAV injections confirmed the OCT findings. In the animal where OCT showed marked focal thinning of the inner retina, we found loss of the RGC and inner nuclear layers with only the outer retina remaining (Figure 6A,B). Other animals did not exhibit such severe inner retinal atrophy, but showed the more characteristic loss of cells in the ganglion cell layer (Figure 6C). In contrast, eyes injected with scAAV-*GFP* showed no loss of the inner retina (Figure 6D,E) or cells in the RGC layer (Figure 6F).

### Postmortem histopathology of optic nerve degeneration

**Figure 7:**
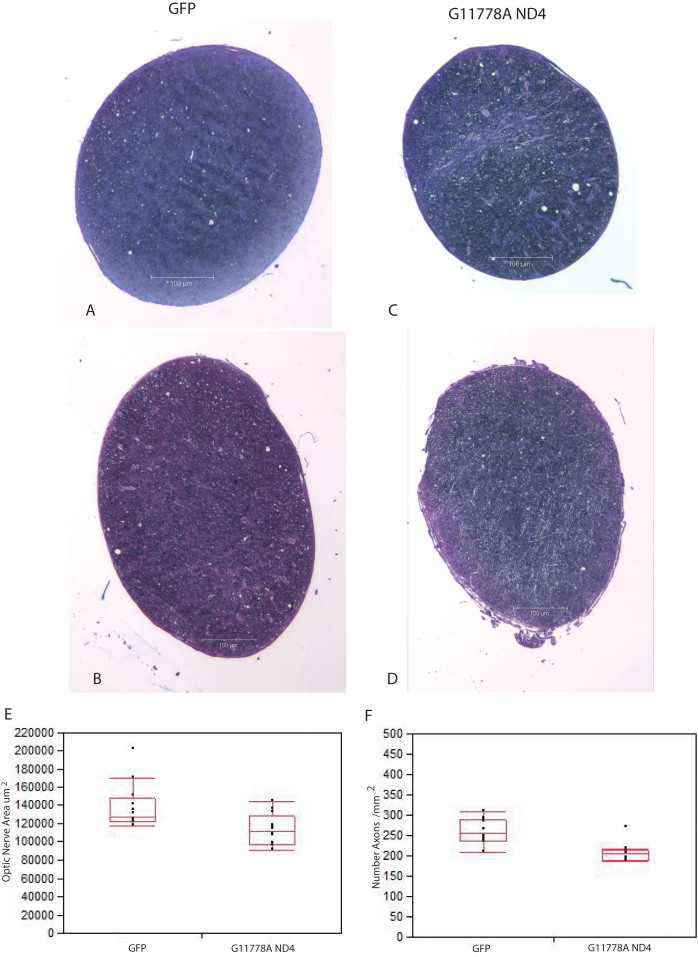
Histopathology of optic nerve atrophy. Relative to the normal optic nerves of contralateral control eyes that received scAAV-*GFP* (**A**, **B**), the hallmark optic nerve atrophy of LHON was prominent in mouse eyes injected with MTS scAAV mutant *ND4* that appeared thinner than the contralateral experimental eyes (**C**, **D**). **E**: A scatterplot with quantile boxes shows quantitation of the optic nerve head areas of COX8 MTS delivered mutant (G11778A) *ND4* were smaller than those of controls injected with scAAV-*GFP*. The lowest line of the quantile plot represents the tenth percentile and the highest line the 90th percentile. The bottom of the quantile box represents the 25th percentile and the top of the box the 75th percentile, with the median value in the middle of the box. Median values are slightly different from the means. **F**: A scatterplot with quantile boxes shows axonal loss in MTS AAV mutant *ND4* injected eyes relative to axon counts in scAAV-*GFP* injected eyes. Values for nine of the mutant *ND4* injected nerves were below the 25th percentile for control optic nerves injected with scAAV-*GFP*.

Postmortem analysis performed 1 year after intravitreal injections confirmed that the MTS-targeted scAAV mutant *ND4* induced the hallmark optic atrophy of LHON. Relative to the normal optic nerves of contralateral control eyes that received scAAV-*GFP* (Figure 7A,B), the hallmark optic nerve atrophy of LHON was prominent in mouse eyes injected with MTS scAAV mutant *ND4 *(Figure 7C,D). Differences in the optic nerve areas between COX8 MTS delivered mutant *ND4* (112,469± 4,981 μm^2^; mean±standard error) relative to AAV-*GFP* (138,300±7,208 μm^2^) were highly significant (p=0.004; Figure 7E). Using transmission electron micrographs to quantitate the number of optic nerve axons, we found that relative to the scAAV-*GFP* injected eyes with a mean of 260±33 (mean±standard deviation) axons per mm^2^, axon counts were significantly reduced in MTS AAV mutant *ND4* injected eyes with a mean of 208±24 axons per mm^2^ (p=0.0004). A quantile plot shows that axon counts of nine mutant ND4 injected optic eyes were below the 25% quantile for the control eyes injected with AAV-*GFP* (Figure 7F). Thus, transmission electron microscopy confirmed that optic atrophy was due to axonal loss.

**Figure 8:**
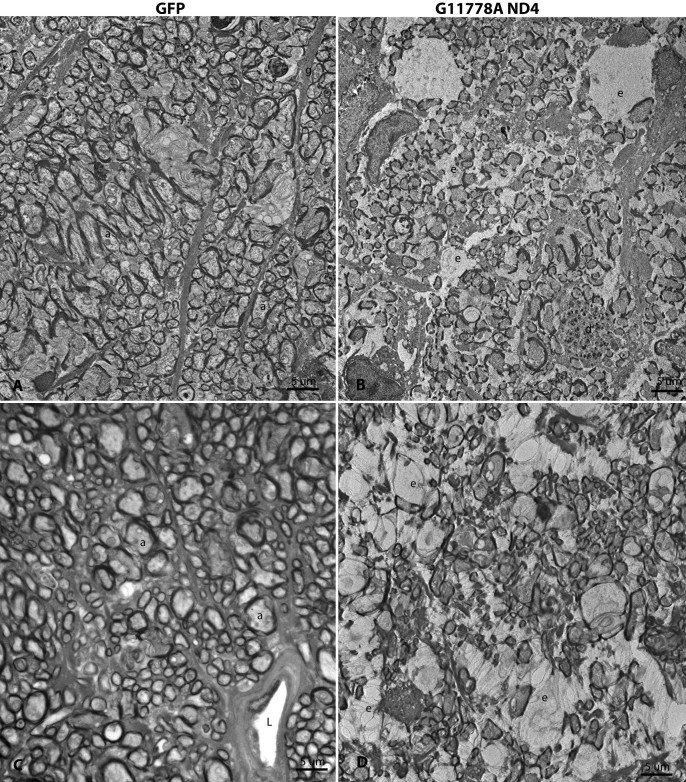
Ultrastructure of optic nerve axonal loss. **A**: One year after injection of scAAV-*GFP*, transmission electron micrographs disclosed optic nerve axons (a) of various sizes enveloped by myelin sheaths. Astroglial cell processes (g) coursed between fibers of the optic nerve. **B**: In this same animal, the opposite eye injected with the MTS scAAV mutant *ND4* had a marked decrease in axonal density. Many empty spaces (e) were present where axons were apparently lost in these atrophic optic nerves and degenerating axonal profiles were evident (d). **C**: In a different animal, the optic nerve of the control eye injected with scAAV-*GFP* had normal axons (a) with the only empty space the lumen of a blood vessel (L). **D**: In this animal, the opposite eye injected with the MTS scAAV mutant ND4 had a marked decrease in axonal density. Many empty spaces (e) were present where axons were lost. a=optic nerve axon, e=empty space, g=astroglial process, d=degenerating axon, L=lumen.

**Figure 9:**
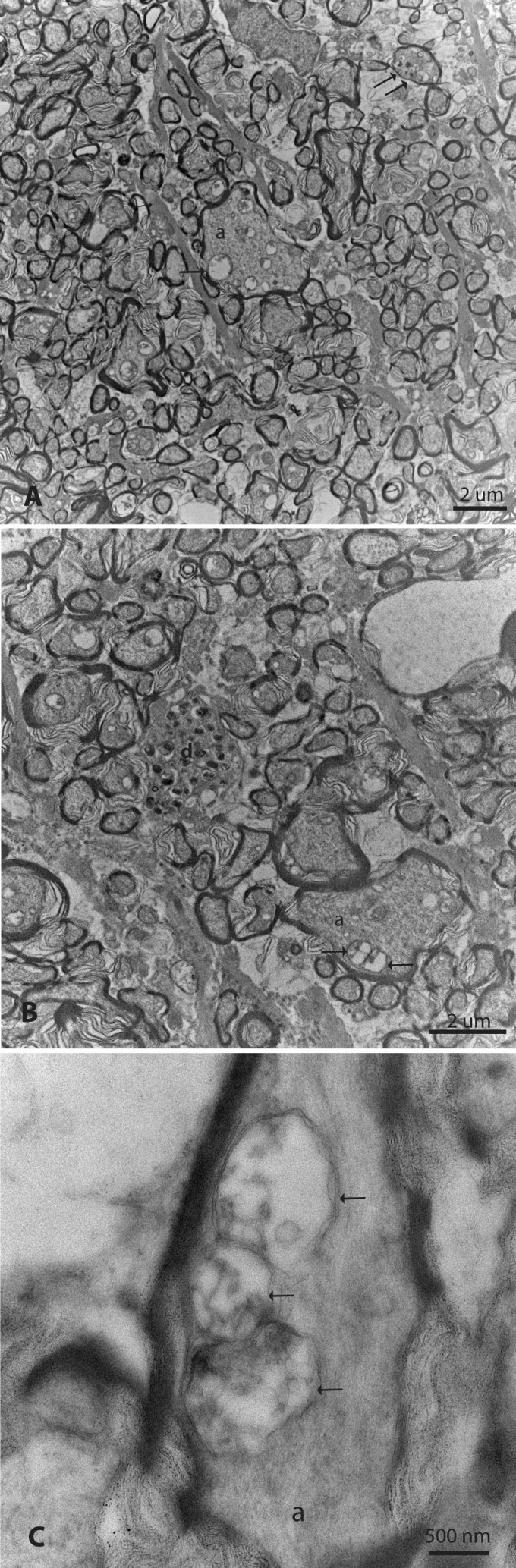
Mitochondria accumulate and fuse. **A**: Optic nerves from mutant *ND4* MTS AAV injected eyes exhibited some large-caliber axons (a) with the accumulation of mitochondria (arrow) of varying diameters. Electron-dense aggregations indicative of irreversible axonal degeneration were also evident (double arrows). **B**: In some swollen axons, two mitochondria were seen fusing (arrows). Irreversible axonal degeneration with intra-axonal electron dense aggregations was evident in other axons (d). **C**: In another axon, three mitochondria (arrows) appeared to fuse.

Ultrastructural analysis of scAAV-*GFP* injected controls revealed a full complement of normal axons enveloped by myelin (Figure 8A). In sharp contrast, axonal density was markedly reduced in the contralateral optic nerve of this same animal, 1 year after injection of scAAV mutant *ND4*, (Figure 8B) with empty spaces present where axons were lost. Ongoing axonal degeneration was still evident in some axons with electron dense aggregations. In a different animal, the control optic nerve injected with AAV-*GFP* had normal axons and myelin (Figure 8C), while the opposite optic nerve injected with scAAV mutant *ND4* showed severe axonal loss with many empty spaces at foci where axons had been (Figure 8D). Even 1 year after the MTS scAAV mutant* ND4* injection, some remaining axons were still in various stages of irreversible degeneration with electron dense aggregations within the axon (Figure 9A,B). Electron lucent mitochondria accumulated in swollen axons (Figure 9A). Fusion of two (Figure 9B) or three mitochondria (Figure 9C) was also evident in axons with otherwise normal appearing neurotubules.

## Discussion

We have shown here that mutant G11778A *ND4* DNA can be introduced into the murine mitochondria of the inner retina, where the expression of the DNA leads to significant mitochondrial perturbations causing loss of visual function, culminating in retinal ganglion cell and optic nerve degeneration that are the hallmarks of human LHON [14,15]. Ocular specimens of autopsied patients with LHON show loss of RGCs and their axons, with the remaining axons showing accumulations of abnormal mitochondria also seen in our animal model system [15]. As human and mouse *ND4* DNA sequences are approximately three-quarters homologous, we excluded a potential adverse consequence of wild-type human *ND4* expression in the murine retina. Using the pattern PERG, a sensitive measure of RGC function, we found no significant differences in amplitude relative to the eyes of normal animals. Thus, unlike the mutant *ND4* allele, rodent visual function did not appear to be altered by the import and expression of the wild-type human *ND4* allele [12]. Nor did we observe retinal ganglion cell loss following intravitreal injection of scAAV-WT-*ND4*.

Although a natural DNA mitochondrial import had been reported in isolated rat liver mitochondria [16] and in plants [17], exogenous DNA cannot be transfected into mitochondria with conventional techniques [18,19].

Our work shows that fusing an AAV capsid protein with a mitochondrial targeting sequence makes an efficient delivery system for importing a relevant gene into the mitochondria of living cells in the mouse visual system where it induced the anticipated characteristic phenotype. Studies by others have conjugated an MTS to exogenous proteins and small linear DNA, which enhanced their delivery to mitochondria [13,20], but that strategy failed in cases of macromolecules and hydrophobic molecules including mtDNA and mitochondrial proteins [21]. Liposome-based carriers can import hybrid molecules into mitochondria of whole cells, but this approach has low efficiency and high cytotoxicity [18,22,23]. Mutated mtDNA can potentially be removed from the germ line by transfer of the spindle-chromosomal complex from one egg to another that is enucleated and depleted of its mitochondria [24]. A major limitation of this successful approach is that it cannot be used after birth, and the technology has not yet been demonstrated to actually exert a mitochondrial phenotype. In contrast, our MTS AAV approach resulted in expression of mutant human *ND4* in mitochondria where it induced visual loss and optic atrophy, thus suggesting that our novel technology may also be useful long after birth for inserting DNA into the mitochondria of live animals.

Animal models of human mitochondrial diseases could be the substrate for testing therapies for human mitochondrial disorders, but these models are scarce, as discussed in a recent review article [25]. These models include mutations introduced into nuclear genes encoding mitochondrial proteins to induce defective oxidative phosphorylation by complex I deficiency [26,27]. A limitation of these mouse model systems is that the defects are rapidly fatal. We took a similar approach by reducing expression of the NDUFAI subunit of complex I [7]. With expression limited to the visual system, this approach induced optic nerve degeneration but was not fatal. That study emphasized the importance of complex I to an organ system, the optic nerve that has a high density of mitochondria.

Since approximately 85% of mitochondrial proteins are encoded in the nucleus, synthesized on cytoplasmic ribosomes, and then imported into the mitochondria under the direction of a mitochondrial targeting sequence, we used “allotopic” expression to import a synthetic *ND4* recoded in the nuclear genetic code that induced a phenotype resembling human LHON in mice [8]. Another group independently suppressed visual loss and RGC demise by adding back in the allotopically expressed wild-type *ND4* allele [28]. The allotopic expression technique developed almost 10 years ago is only now under investigation for human LHON clinical trials [12]. Still, a limitation of the allotopic expression technology is that it appears to be highly dependent on the MTS, the recoded mitochondrial gene, and the appended epitope tag used for immunodetection [1]. Moreover, allotopic expression is useful only for expressing the 13 proteins encoded by mitochondrial DNA. Our technology for delivering DNA to mitochondria is useful for generating relevant animal models for these disorders as well as other disorders such as mitochondrial encephalomyopathy, lactic acidosis, and stroke-like episodes or myoclonic epilepsy and ragged red fibers caused by mutations in tRNAs encoding mitochondrial genes or others disorders such as Kearns-Sayre syndrome and chronic progressive external ophthalmoplegia caused by large deletions in the mitochondrial genome [29].

Using an MTS to direct a virus to deliver a mitochondrial gene to the organelle may be more versatile. Rather than introducing a mutant mitochondrial gene, MTS AAV may also be useful for introducing the wild-type allele into patients with mutated mitochondrial DNA. We anticipate that DNA encoding tRNAs or deletions in the mitochondrial genome may also be shuttled into the mitochondria with MTS AAV technology. As shown here, the technology can be extremely useful for creating a bona fide animal model of LHON with the desired phenotype in the target organ system. Relevant animal model systems with mutated mitochondrial DNA are highly relevant for testing potential therapies for the many diseases caused by mutated mitochondrial DNA. We look forward to providing this delivery system to other laboratories for introducing other mutant mitochondrial genes by injecting MTS AAV into other organ systems such as the brain, heart, or skeletal muscle of animals and eventually for the benefit of the multitude of patients afflicted by disorders caused by mutated mitochondrial DNA for whom there is currently no effective remedy.
